# Rearranged During Transfection Rearrangement Detection by Fluorescence In Situ Hybridization Compared With Other Techniques in NSCLC

**DOI:** 10.1016/j.jtocrr.2024.100714

**Published:** 2024-08-29

**Authors:** Anne Mc Leer, Julie Mondet, Nelly Magnat, Mailys Mersch, Diane Giovannini, Camille Emprou, Anne-Claire Toffart, Nathalie Sturm, Sylvie Lantuéjoul, David Benito

**Affiliations:** a Université Grenoble Alpes, Grenoble, France; b Service d’Anatomie et Cytologie Pathologiques, Pôle de Biologie et Pathologie, CHU Grenoble Alpes, Grenoble, France; c Institute for Advanced Biosciences, Université Grenoble Alpes, Grenoble, France; d TIMC-IMAG, Université Grenoble-Alpes, La Tronche, France; e Clinique Hospitalo-Universitaire de Pneumologie Physiologie, Pôle Thorax et Vaisseaux, CHU Grenoble Alpes, Grenoble, France; f Cancer Research Center Lyon, Centre Léon Bérard, Lyon, France; g Medical Affairs - Oncology, Eli Lilly (Suisse) S.A., Dubai, United Arab Emirates

**Keywords:** RET, Fusion gene, Lung cancer, FISH, RNA-sequencing, Targeted therapy

## Abstract

**Introduction:**

*RET* rearrangements occur in 1% to 2% NSCLCs. Since no clinically validated RET antibody is currently available, fluorescence in situ hybridization (FISH) is often used as a screening tool to identify patients likely to benefit from RET-targeted therapy. In this study, we performed a comprehensive review of publications in which *RET*-rearrangement testing was performed by FISH and compared the methods and results with our data.

**Methods:**

The findings of an electronic search for publications using *RET*-FISH in lung cancer were compared with the results obtained at the Grenoble University Hospital where 784 *EGFR-*, *KRAS-*, *ALK*-, and *ROS1*-negative NSCLCs were tested by *RET* break-apart FISH and confirmed by RNA-sequencing (RNA-seq).

**Results:**

Out of the 85 publications using *RET*-FISH analysis, 52 pertained to patients with lung cancer. The most often used positivity threshold was 15%. Six publications compared *RET*-FISH with at least one other molecular technique on at least eight samples, and the concordance was variable, from 5.9% to 66.7% for FISH-positive cases. Regarding our data, out of the 784 analyzed samples, 32 (4%) were positive by *RET*-FISH. The concordance between *RET*-FISH and RNA-seq in *RET*-FISH positive samples was 69%.

**Conclusions:**

Overall, both existing literature and our data suggest that *RET*-FISH testing can be used for rapid screening of *RET* rearrangements in NSCLC. Nevertheless, using an orthogonal technique such as RNA-seq to confirm *RET*-FISH-positive cases is essential for ensuring that only patients likely to benefit from *RET*-target therapy receive the treatment.

## Introduction

Kinase gene fusions are the product of chromosomal rearrangements, an important class of oncogenic drivers associated with many solid tumors and hematologic malignancies.^1^, ^2^, ^3^, ^4^ The *RET* gene (located at chromosome 10q11.21) encodes a single-pass transmembrane tyrosine kinase receptor. Under normal circumstances, this receptor interacts with its ligands by means of glial cell-line-derived neurotrophic factor family receptor-α co-receptors and mediates cellular processes such as proliferation, differentiation, survival, migration, and metabolism, playing important roles in the development and maintenance of the enteric nervous and genitourinary systems, and various other tissue types, such as the nervous and neuroendocrine tissues.^5^ Fusions involving the RET receptor tyrosine kinase are created by the in-frame chromosomal fusion of the 3′ tyrosine kinase domain of the *RET* proto-oncogene to the 5′ regions of various heterologous partner genes. These fusions have been identified in various cancers,^6^ but occur predominantly in non-medullary thyroid carcinomas (TCs), including 10% to 20% of all papillary TCs and to a lesser extent in follicular TCs, and anaplastic (undifferentiated) TCs.^7^, ^8^, ^9^ *RET* gene fusions also occur in 1% to 2% of NSCLCs, mainly in adenocarcinomas.^10^, ^11^, ^12^, ^13^, ^14^ Targeted therapy with multikinase inhibitors has shown modest clinical activity, with objective response rates ranging from 0% to 50% in patients with NSCLC with *RET* fusions,^15^ lower than the rates obtained with ALK and ROS1 small molecule inhibitors (up to 83 and 77%, respectively).^16^^,^^17^ Nevertheless, in early-phase clinical trials, novel selective RET inhibitors, such as LOXO-292 (selpercatinib) and BLU-667 (pralsetinib) revealed objective response rates of 61%^18^ and 64%,^19^ respectively, in pretreated patients with NSCLC, and of 84%^18^ and 72%,^19^ respectively, in patients who were treatment-naive. LOXO-292 and BLU-667 have been approved since 2020 by the American Food and Drug Administration for the treatment of advanced RET-driven NSCLC, and other potent and selective RET inhibitors, such as BOS172738^20^ and KL590586 (NCT05265091) are undergoing clinical evaluation. In addition, *RET* rearrangements have been reported as a resistance mechanism in patients with *EGFR*-mutated NSCLC treated with tyrosine kinase inhibitors.^21^

Collectively, these data highlight the importance of implementing robust and practical screening methods to identify patients who are likely to benefit from RET-targeted therapy.

In many Pathology laboratories, fluorescence in situ hybridization (FISH), is often used for screening of *RET* gene rearrangements in patients with NSCLC, since RET immunohistochemistry (IHC) shows low sensitivity and specificity.^21^, ^22^, ^23^, ^24^ The turn-around time of the technique is short (1–2 d) and small amounts of tissue are needed.

In this study, we performed an up-to-date comprehensive review of publications in which FISH was used to detect *RET* rearrangements to understand the testing environment and the potential utility of this technique when compared with other molecular diagnostic tools. We then contrasted the results obtained with our own data.

## Materials and Methods

### Literature Review

We performed a systematic literature review by 'pearl growing', citation chasing, and PubMed search for studies published between 2000 and 2022 mentioning *RET*-FISH in their methodology. A total of 86 publications were identified (Supplementary Table 1), and out of these, 52 were lung cancer-related, the rest concerned other tumor types, such as TC (Supplementary Table 2).

### Patients

From February 2013 to February 2021, 784 specimens of primary NSCLC were sent to the Grenoble University Hospital cancer molecular genetics platform for routine lung cancer biomarker testing. These specimens were either formalin- or AFA-fixed (the nature of the fixative was not systematically specified), and paraffin-embedded. They included small biopsies (bronchial, transthoracic, or liver biopsies) and surgical specimens (lung resections, lymph node, pleural, or pericardial surgical biopsies). All 784 specimens were tested at least for *EGFR* and *KRAS* mutations and immunohistochemical expression of ALK and ROS1 proteins, and were all negative.

This study was conducted according to the European General Data Protection Regulation. The data used are derived from an aggregated, non-individualized database. No personal data allowing identification of subjects was used in this work.

### *RET*-FISH

FISH was performed on unstained 4 μm formalin- or AFA-fixed paraffin-embedded tumor tissue sections with the use of a *RET* break-apart probe set (ZytoLight SPEC RET Dual Color Break Apart Probe, ZytoVision, Clinisciences, France) using a paraffin pretreatment reagent kit (Vysis, Abbott Molecular or Dako, Agilent Technologies, France). Assays were performed following the manufacturers’ instructions. Nuclei were counterstained with 4',6-diamidino-2-phenylindole-Vectashield (Vektor Laboratories, Ab-Cys, Paris, France). Sections were analyzed with a GSL10 Leica slide scanning system (Leica, France) under a 63× oil immersion objective with a fluorescence microscope equipped with appropriate filters, a charge-coupled device camera, and the FISH imaging and capturing software CytoVision (Leica Biosystems, Nanterre, France). Signals were enumerated with the CytoVision software (Leica Biosystems). Non-rearranged (negative) *RET*-FISH revealed fusion signals or very close apposition of the probes adjacent to the 5′ (orange) and the 3′ (green) ends of the gene. Rearranged *RET*-FISH appeared as split 3′ and 5′ (with a gap between the 5′ and 3′ signals being greater than the largest of the two signal diameters), or isolated 3′ (green) signals. Tumor tissues were considered *RET*-FISH positive (*RET*-FISH rearranged) if at least 15% of tumor cells were positive in at least 60 tumor cells, on the basis of the criteria used for *ALK* FISH.^25^ Samples with less than 60 analyzable tumor cells were considered not interpretable. Otherwise, the samples were considered as being *RET*-FISH negative.

All *RET*-FISH positive samples were checked by targeted RNA-sequencing (RNA-seq) for the presence of a *RET* fusion transcript.

### Targeted RNA-Seq and Data Analysis

Library preparation was performed using either the RNA Fusion Lung Cancer panel from 10 ng of total RNA (Thermo Fisher Scientific, Illkirch, France) or the FusionPlex Lung panel from 200 ng of total nucleic acids (ArcherDx, Boulder, Colorado), following the manufacturers’ instructions. Libraries were sequenced on a Thermo Fisher sequencer (Ion PGM or Ion S5). The latest versions of the Ion Reporter (Thermo Fisher Scientific) or Archer Analysis (ArcherDx) software were used to identify fusion gene products from raw sequence data.

## Results

### Literature Review Findings

#### Reported Concordance Between RET-FISH And Other Molecular Techniques

**Figure 1:**
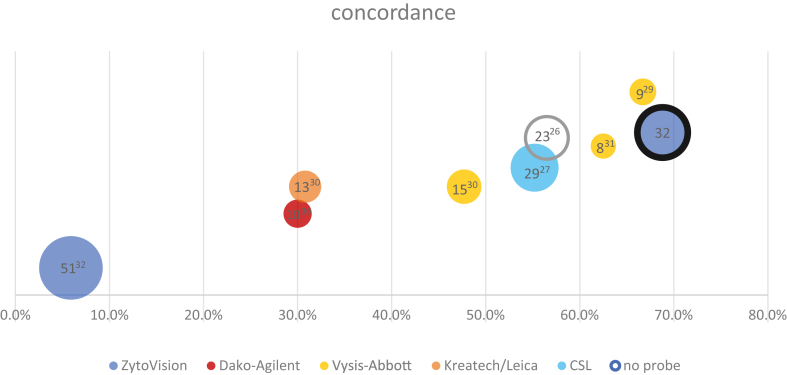
Concordance values between FISH and another molecular technique. On this graph, bubble size is the number of *RET*-FISH+ samples compared with another technique. The highlighted bubble represents our data (local cohort). The concordance values are based on the data in Table 1. FISH, fluorescence in situ hybridization.

Out of the 52 publications identified using FISH for *RET*-rearrangement detection in lung cancers, 31 studies reported positive cases by break-apart FISH, which were also tested by at least one other molecular technique (mostly reverse transcription polymerase chain reaction [RT-PCR], next-generation sequencing [NGS], or NanoString) (Tables 1 and 2).^11^^,^^14^^,^^22^^,^^23^^,^^26^, ^27^, ^28^, ^29^, ^30^, ^31^, ^32^, ^33^, ^34^, ^35^, ^36^, ^37^, ^38^, ^39^, ^40^, ^41^, ^42^, ^43^, ^44^, ^45^, ^46^, ^47^, ^48^, ^49^, ^50^, ^51^, ^52^, ^53^ Studies that did not compare *RET*-FISH positive cases with another molecular technique, for which comparison data were incomplete, or which were case reports, were not included. In nine of these 31 studies, *RET*-FISH was used as the initial method or screening method and the results were compared with at least one other molecular technique, leading to very variable concordances, and sometimes very few samples (Table 1).^11^^,^^14^^,^^22^^,^^23^^,^^26^, ^27^, ^28^^,^^30^, ^31^, ^32^, ^33^, ^34^, ^35^, ^36^, ^37^, ^38^, ^39^, ^40^, ^41^, ^42^, ^43^, ^44^, ^45^, ^46^, ^47^, ^48^, ^49^, ^50^, ^51^, ^52^, ^53^ For the six studies where FISH was used as the screening method and the concordance with another technique was assessed on more than eight samples,^26^^,^^27^^,^^30^, ^31^, ^32^^,^^53^ the concordances varied from 5.9% to 66.7% for FISH-positive cases (Table 1^11^^,^^14^^,^^22^^,^^23^^,^^26^, ^27^, ^28^^,^^30^, ^31^, ^32^, ^33^, ^34^, ^35^, ^36^, ^37^, ^38^, ^39^, ^40^, ^41^, ^42^, ^43^, ^44^, ^45^, ^46^, ^47^, ^48^, ^49^, ^50^, ^51^, ^52^, ^53^ and Fig. 1). The rest of the studies (22) used FISH as a validation technique for RT-PCR, NanoString or NGS assays. Notably, one additional study reported two cases that were not conclusive by FISH but were also found not conclusive by NanoString because of pre-analytical issues.^54^

**Table 1:** Concordance Between *RET*-FISH and Other Molecular Techniques Performed on Lung Cancer Reported in the Literature: Cases Initially Screened by FISH

Study	Number of *RET*-FISH+ Samples Reported	Number of *RET*-FISH+ Samples Compared With Other Techniques	Number and % of Concordant Samples Between FISH and Other Techniques	Comments
Kim et al.,^32^ 2018	51	51	3 (5.9) [NanoString]	
Radonic et al.,^30^ 2021	48	30	9 (30) [RNA-seq]	
Tsuta et al.,^27^ 2014	50	29	16 (55.2) [RT-PCR]	RT-PCR analysis only when RNA was available (29) and 14 *KIF5B::RET* fusions and 2 *CCDC6::RET* fusions were confirmed.
Takeuchi et al.,^26^ 2012	22	22	12 (54.5) [RT-PCR]	
Tan et al.,^29^ 2020	30	9	6 (66.7) [NGS]	2 equivocal FISH samples (10-15% positive cells) were also positive by RNA-seq.
Baker et al.,^31^ 2021	8	8	5 (62.5) [RNA-seq]	
Go et al.,^33^ 2013	3	3	3 (100) [PCR] 3 (100%) [WTS]	
Rogers et al.,^28^ 2017	1	1	0 (0) [NanoString] 1 (100) [Agena] 0 (0) [RNA-seq]	The only *RET*-FISH-positive case in this study was also the most degraded sample, failing to be detected by NanoString and ThermoFisher RNA-seq, and was borderline positive with Agena allele-specific assay.
Piton et al.,^34^ 2018	1	1	1 (100) [ligation-dependent RT-PCR]	FISH-positive cases with rearranged nuclei between 15 and 20% were excluded because it was a high risk of a false-positive result, as the authors did not want to test LD-RT-PCR on these unsure 'positive' cases.

FISH, fluorescence in situ hybridization; LD-RT-PCR, ligation-dependent reverse transcription polymerase chain reaction; NGS, next generation sequencing; PCR, polymerase chain reaction; RNA-seq, RNA-sequencing; RT-PCR, reverse transcription polymerase chain reaction; WTS, whole transcriptome sequencing.

**Table 2:** Concordance Between *RET*-FISH and Other Molecular Techniques Performed on Lung Cancer Reported in the Literature: Cases Initially Screened by Another Method Where FISH Was Used as a Confirmatory/Validation Technique for the Chosen Screening Technique

Study	Number of *RET*-FISH+ Samples Reported	Number of *RET*-FISH+ Samples Compared With Other Techniques	Number and % of Concordant Samples Between FISH and Other Techniques
Takeuchi et al.,^36^ 2021	34	34	4 (100) [RT-PCR] 30 (100) [RNA-seq]
Yang et al.,^23^ 2021	27 lung cancers (subset of the 171 samples with a *RET* structural variant)	27	27 (100) [DNA-NGS] 25 (88.9) [RNA-seq]
Feng et al.,^49^ 2022	25	25	25 (100) [RNA-seq]
Shang et al.,^47^ 2019	20	20	20 (100) [RT-qPCR]
Yoh et al.,^51^ 2017	19	19	19 (100) [RT-PCR]
Lira et al.,^45^ 2014	15	15	15 (100) [NanoString]
Pan et al.,^39^ 2014	15	15	15 (100) [RT-PCR]
Lee et al.,^22^ 2015	14	14	14 (100) [NanoString]
Wang et al.,^14^ 2012	13	13	13 (100) [RT-PCR]
Song et al.,^37^ 2016	11	11	11 (100) [RT-PCR] 11 (100) [RNA-seq]
Chen et al.,^48^ 2020	10	10	10 (100) [DNA-NGS]
Radonic et al.,^30^ 2021	9	9	9 (100) [RNA-seq]
Kim et al.,^40^ 2015	9	9	9 (100) [RT-PCR]
Kohno et al.,^11^ 2012	6	6	6 (100) [RT-PCR]
Tanaka et al.,^43^ 2017	4	4	4 (100) [RT-PCR]
Sokolova et al.,^52^ 2020	3	3	3 (100) [validated method]
Sasaki et al.,^35^ 2012	2	2	2 (100) [RT-PCR]
Reguart et al.,^42^ 2017	2	2	2 (100) [NanoString]
Song et al.,^41^ 2017	2	2	2 (100) [RT-PCR]
Ambrosini-Spaltro et al.,^50^ 2022	2	2	2 (100) [RNA-seq]
Suehara et al.,^44^ 2012	1	1	1 (100) [NanoString]
Borrelli et al.,^38^ 2013	1	1	1 (100) [RT-PCR]
Velizheva et al.,^46^ 2018	1	1	1 (100) [RNA-seq]

DNA-NGS, DNA next generation sequencing; FISH, fluorescence in situ hybridization; RNA-seq, RNA-sequencing; RT-PCR, reverse transcription polymerase chain reaction; RT-qPCR, reverse transcription quantitative polymerase chain reaction.

#### *RET*-FISH Probes And Methodology Used In The Literature

A wide range of probes was used across publications (Supplementary Table 2), targeting various lengths and locations within the 5′ and 3′ regions of the *RET* gene. Out of the 52 publications, 40 mentioned the use of commercial break-apart probes (one publication mentioned four probes^30^). Among the commercial probes (provided by 12 different manufacturers), the most often used (39%) was the ZytoLight SPEC *RET* Dual Color Break (ZytoVision GmbH) (Supplementary Table 3).

#### Scoring

##### Signal Patterns

Regarding the scoring, positivity was considered when only a separation of the 5′ and 3′ signals (split signal) was found, or when a split signal or single 3′ signals or both, were present by an equal proportion of authors (Supplementary Table 4). The split signal was considered positive when the gap between the 5′ and 3′ signals was either at least more than one signal diameter or in some cases, a separation greater than twice the signal diameter.^23^^,^^55^ In general, a “complex” or “atypical” pattern was defined as a rearrangement with any pattern that could not be classified using the usual split or single 3′ patterns. Some authors considered complex or atypical patterns as potentially positive, and a confirmatory test was always initiated if sufficient tissue was available. Single 5′ patterns were reported sometimes but were mostly considered clinically negative, because of the potential loss of the RET kinase domain.^30^ Notably, one publication^38^ considered single 5′ *RET* signals to be positive.

##### Cutoff Value

**Figure 2:**
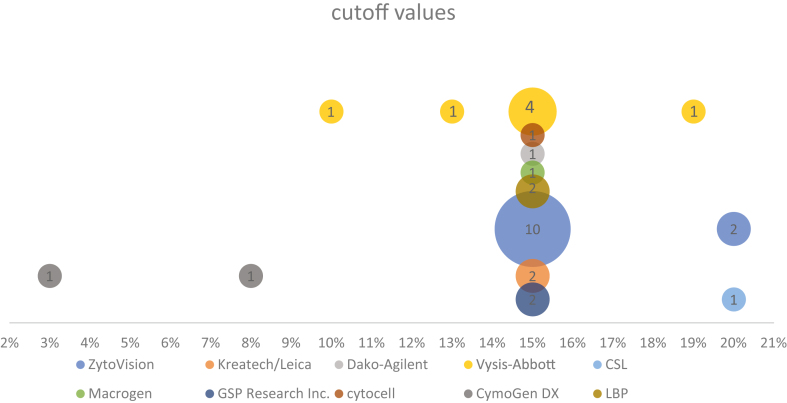
*RET*-FISH cutoff values used in the literature. Graph showing the number of times a cutoff value was mentioned in the literature for *RET*-FISH (31 publications identified). Bubble size is the number of mentions for that probe (33 mentions in total). FISH, fluorescence in situ hybridization.

Since there are no standard guidelines to date regarding the cutoff value for *RET*-FISH positivity in lung tumors, we found several approaches and different cutoff values cited in the literature, ranging from 3% to 20% of cells with *RET*-positive patterns. Nevertheless, as shown in Figure 2, in most studies a threshold of at least 15% FISH-positive tumor nuclei was chosen to define a *RET*-FISH-positive tumor. In other studies, to consider a sample positive for a *RET*-rearrangement, the cutoff was determined as the mean value of positive cells +3 S.D. in known *RET*-negative^31^^,^^56^ or *RET*-positive samples.^55^^,^^57^ Remarkably, 20 articles of 52 did not specify the cutoff used.

### *RET* Testing Results on Our NSCLC Cohort

#### *RET*-FISH Results

**Figure 3:**
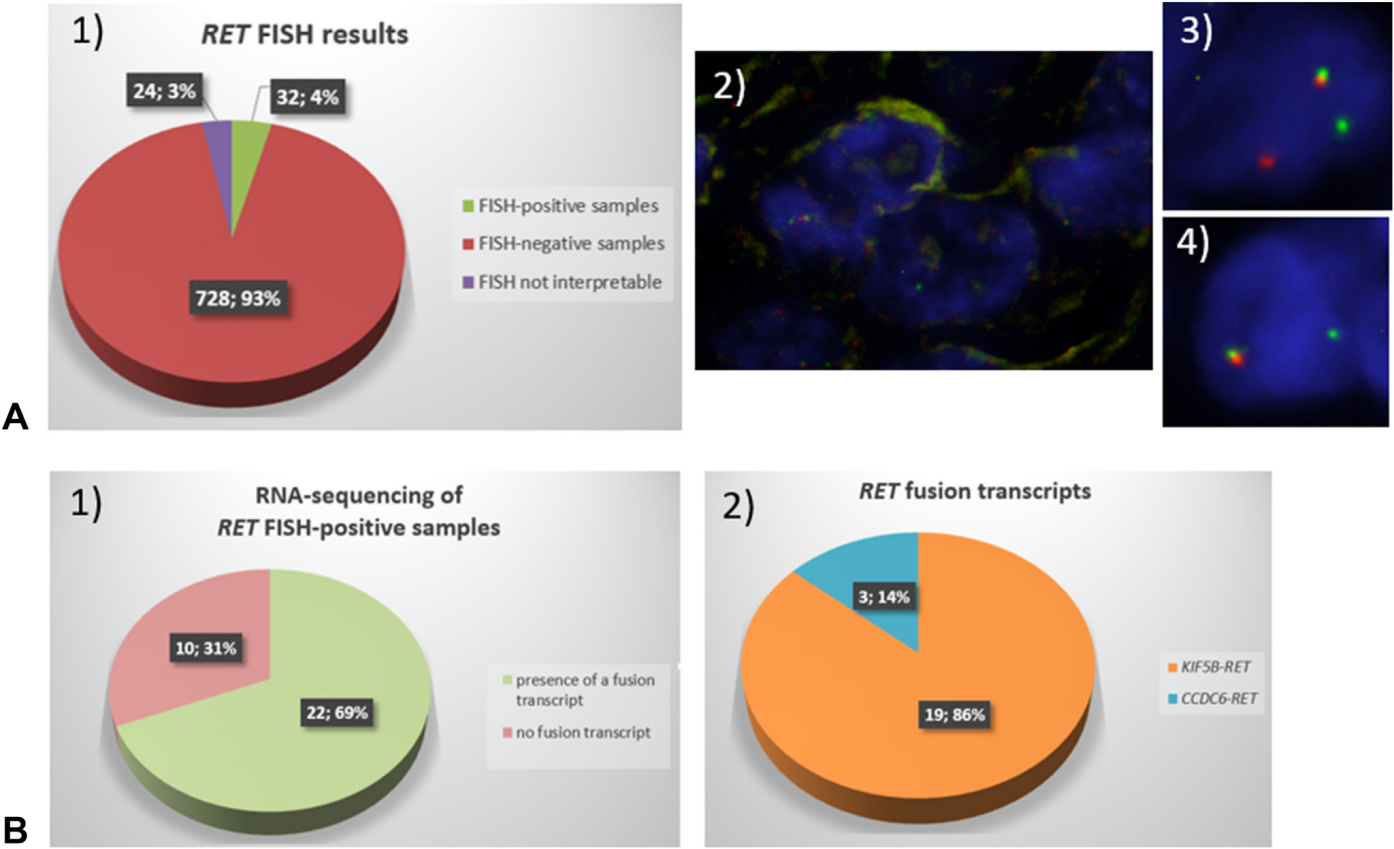
RET-FISH results on the 784 NSCLC samples analyzed and targeted RNA-seq results on the 32 RET-FISH positive samples. *(A)* RET-FISH results on the 784 NSCLC samples of our NSCLC cohort. (1) Repartition of the RET-FISH positive, negative, and not interpretable samples. (2) Example of a non-interpretable result (signal intensity too low), (3) Example of a RET-FISH positive nucleus showing a split signal, (4) Example of a RET-FISH positive nucleus showing an isolated 3′ (green) signal. *(B)* Targeted RNA-seq results on the 32 RET-FISH positive samples. (1) Number and proportion of RET-FISH positive cases for which a fusion transcript was present or absent. (2) Nature and repartition of the fusion transcripts detected. FISH, fluorescence in situ hybridization.

Out of the 784 samples analyzed by *RET*-FISH, 24 (3%) were not interpretable, either because of the absence or very poor quality of the hybridization signals (16 samples) or because less than 60 analyzable tumor cells were present on the tissue section analyzed (eight samples) (Fig. 3). Out of the 760 samples for which the *RET*-FISH analysis yielded an interpretable result, 32 (4%) were positive by FISH (≥15% of tumor cells with separated 5′ and 3′ signals or isolated 3′ signals or both) and 728 / 760 (96%) were negative (<15% positive tumor cells) (Fig. 3 and Table 3). Notably, no samples harbored a FISH pattern showing isolated 5′ (orange) signals. Nineteen patients were tested twice: 13 patients were analyzed at diagnosis and then at progression 1 to 3 years later, five patients had *RET*-FISH analysis performed first on a biopsy (n = 3) or a cytologic specimen (n = 2) and then on a resection sample, and one patient had two biopsies from two different metastatic sites. For all 19 patients, *RET*-FISH analyses were concordant: negative on all samples.

**Table 3:** Clinical, Histopathologic, and FISH Data of the 760 *RET*-FISH-Positive and -Negative NSCLC Samples

N = 760	*RET*-FISH-Positive Samples (≥15%) n = 32	*RET*-FISH-Negative Samples (<15%) n = 728
Patient gender, n (%)		
M	18 (56)	493 (68)
F	14 (44)	235 (32)
Patient age, mean, median (range)		
M	70, 71 (51–86)	68, 69 (21–94)
F	67, 65 (48–82)	66, 66 (29–93)
Histology, n (%)		
Adenocarcinoma	24 (75)	583 (80.1)
Squamous cell carcinoma	0	7 (1.0)
Sarcomatoid carcinoma	0	3 (0.4)
(large cell) neuroendocrine carcinoma	1 (3.1)	6 (0.8)
Other type of carcinoma	7 (21.9)	129 (17.7)
Type of sample, n (%)		
Lung resection	7 (21.9)	123 (16.9)
Resection of metastatic site	4 (12.5)	70 (9.6)
Lung biopsy	13 (40.6)	344 (47.3)
Biopsy of metastatic site	5 (15.6)	132 (18.1)
Cytologic sample	3 (9.4)	59 (8.1)
% of tumor cells in the analyzed sample, n (%)		
<20%	5 (15.6)	86 (11.8)
20%–50%	20 (62.5)	434 (59.6)
>50%	7 (21.9)	203 (27.9)
Not specified	0	5 (0.7)
Number of nuclei analyzed by FISH, mean (range)	105 (84–128)	105 (60–311)
% of *RET*-FISH positive nuclei mean, median (range)	55.3%, 57.7% (17.7%–94%)	4.4%, 3.9% (0–14.3%)

F, female; FISH, fluorescence in situ hybridization; M, male.

The mean and median percentages of FISH-positive cells in the *RET*-FISH-negative samples were 4.4% and 3.9%, respectively, ranging from 0% to 14.3% (Table 3). The sample with 14.3% positive tumor cells was analyzed by RNA-seq and revealed no fusion transcript. The mean and median percentages of FISH-positive cells in the *RET*-FISH-positive samples were 55.3% and 57.7%, respectively, ranging from 17.7% to 94% (Table 3).

#### Targeted RNA-seq Results

All 32 samples showing a *RET*-FISH positive result were analyzed by RNA-seq using targeted panels from Thermo Fisher Scientific or ArcherDx or both. As shown in Figure 3, a *RET* fusion transcript was detected in 22 (69%) samples. In three (14%) cases, a *CCDC6::RET*(exon 12) transcript was found (*CCDC6* exon 1 in two cases, exon 8 in one case), and in all other positive cases (19, 86%), a *KIF5B::RET* fusion transcript was found. All *KIF5B::RET* fusions were between exon 15 of *KIF5B* and exon 12 of *RET*, except for one sample for which the fusion was within intron 11 of *RET*.

#### Comparison Between FISH And RNA-seq Results For RET-FISH Positive Samples

When analyzed in detail, the FISH patterns between the 22 *RET*-FISH positive and RNA-seq positive samples and the 10 *RET*-FISH positive and RNA-seq negative samples appeared to be somewhat different (Table 4). Indeed, in the FISH and RNA-seq discordant samples, the isolated 3′ signal pattern was more represented (median of 86% of positive nuclei) than in the FISH and RNA-seq concordant samples (37%). Conversely, the split pattern was more represented in the FISH and RNA-seq concordant samples (median of 63%) than in the FISH and RNA-seq discordant samples (14%).

**Table 4:** Concordance Between *RET*-FISH and *RET*-RNA-seq in the 32 *RET*-FISH-Positive NSCLC Samples

N = 32	*RET*-FISH Positive/RNA-seq Positive Samples n = 22	*RET*-FISH Positive/RNA-seq Negative Samples n = 10
% of positive cells showing a split pattern, median	62.9	13.7
% of positive cells showing an isolated 3′ signal pattern, median	37.1	86.3

FISH, fluorescence in situ hybridization; RNA-seq, RNA-sequencing.

The 10 *RET*-FISH positive and RNA-seq negative samples were checked twice, either by two different RNA-seq panels (ThermoFisher and Archer Dx) or by RNA-seq and real-time RT-PCR for *KIF5B*::*RET* and *CCDC6::RET* fusions, if not enough material was available for another round of RNA-seq. All 10 cases were negative by all these RNA-based techniques, pointing toward a false-positivity of the *RET*-FISH analysis of 31%.

## Discussion

Since the approval of RET inhibitors by the United States Food and Drug Administration for the treatment of *RET* fusion-positive tumors, the accurate identification of *RET* rearrangements has gained predictive significance. Optimizing diagnostic tools for sensitivity and specificity is therefore essential but these techniques must also be adapted to the low prevalence of *RET* fusions in NSCLC. Historically, FISH has been the reference technique used for rearrangements and fusions detection in solid tumors, but several other diagnostic tools have been developed, such as IHC, RT-PCR, and NGS techniques. Nevertheless, even if RET antibodies are available, they do not have the sensitivity/specificity to be clinically relevant for the detection of RET fusion proteins, and International Association for the Study of Lung Cancer/College of American Pathologists/Association for Molecular Pathology Guidelines recommend against using IHC to test for *RET* fusions in patients with lung cancer.^58^ PCR-based methods, such as RT-PCR and amplicon-based NGS can be limited by the set of primers available for each assay, potentially missing novel fusion partners or atypical breakpoints, and NGS techniques on the basis of hybrid capture or multiplex anchored PCR can detect *RET* fusions regardless of the fusion partner, but are not necessarily available in every laboratory. Therefore, FISH continues to be used as a screening method in some instances, for example when testing *RET* in a sequential algorithm, especially in *EGFR*-, *KRAS*-, *ALK*- and *ROS1*-negative NSCLC samples and/or when NGS or RT-PCR are not available or technically feasible. FISH turn-around time is short (1–2 d) and requires small amounts of tissue. Among the various *RET*-FISH probes available, break-apart probes are more suited to the detection of *RET* rearrangements compared with fusion probes since nearly 50 *RET* fusion partners have been identified in NSCLC.^59^

In the present report, we aimed to compare our data with the available published data from 2000 to 2022 regarding *RET*-FISH testing to understand the testing environment and to analyze the advantages and drawbacks of *RET*-FISH compared with other molecular testing methods.

In the absence of validated interpretation criteria for the detection of *RET* rearrangements by break-apart FISH, we applied criteria extrapolated from *ALK* FISH testing^60^^,^^61^ and found them comparable to those used by most of the published studies. Indeed, out of the 52 publications reporting the use of FISH for *RET*-rearrangement detection in lung cancer, when mentioned, the most often used positivity threshold (cutoff value) was 15%, and the FISH patterns considered positive in most studies were split signals (one to two signal diameters apart) and isolated 3′ signals. With respect to the FISH probes used, we found an impressive variety (>10) of commercial probes, but the most largely used was the ZytoVision *RET* break-apart probe.

Regarding the positivity thresholds applied, in the study by Michels et al.,^62^ two different positivity thresholds (either 15% or 20%) were used by the two centers participating in the study, and no differences were found, as the 22 *RET*-FISH positive cases were all found to have more than 20% tumor cells with the ZytoVision *RET* break-apart probe, and the mean percentage of positive cells (fraction of *RET* rearranged cells) in the positive cases was 47.9%, largely above the two positivity thresholds. Unfortunately, in this study, the authors did not compare the FISH results with another technique. In another study by Baker et al.,^31^ in which various positivity thresholds were tested, by using training and validation sets of both NSCLC and non-medullary thyroid cancers, the authors proposed for Abbott’s Vysis *RET* break-apart probe a three-tiered scoring system aiming at maximizing sensitivity given the small number of *RET* fusion-positive cases. Samples with less than 13% interpretable tumor nuclei with abnormal signal patterns were considered negative, samples harboring 13% to 18% abnormal signal patterns were equivocal, needing to be checked by another technique, and samples with 19% or more nuclei with an abnormal signal pattern were considered positive. This 3-tiered system led to 100% sensitivity and 96% specificity of *RET* break-apart FISH on a validation set consisting of 96 samples, out of which were 14 NSCLC *RET* fusion-positive samples confirmed by NGS. Notably, two samples that were equivocal or negative by FISH (13% and 18% of rearranged tumor nuclei) were found to be positive by NGS, with *KIF5B* as the fusion partner, pointing toward the importance of checking both borderline positive and borderline negative samples.

When studying the articles evaluating the concordance between *RET*-FISH and at least one other molecular diagnostic technique (excluding RET IHC and flow cytometry) on eight or more *RET*-FISH positive samples, we found highly variable concordance rates, ranging from 5.9% to 66.7%. Many reasons can explain this variability in concordance rates, especially the wide variety of FISH probes, interpretation criteria, and the comparison methods used (various DNA-based and RNA-based NGS panels, (RT)-PCR, NanoString, Agena, among others). In our laboratory, by using *RET*-FISH as a screening method for *RET*-rearrangement detection on 784 *EGFR*-, *KRAS-*, *ALK*- and *ROS1*-negative NSCLC samples, we found 32 samples to be positive by FISH with the previously mentioned positivity criteria, making our study the second largest study of *RET*-FISH positive samples to date after the study by Kim et al.^32^ Nevertheless, in this latter study, a fusion transcript was identified by NanoString analysis in only three samples leading to a very low concordance (6%) between the two techniques. In contrast, after performing RNA-seq on all 32 *RET*-FISH-positive samples of our cohort to check for the presence or absence of a *RET* fusion transcript, we found a *RET* fusion transcript in 22 samples, showing a concordance of 69% between FISH and RNA-seq. Therefore, if we compare our concordance rate to those of the 6 previous studies we found in the literature which used *RET*-FISH and then confirmed the results on more than 8 samples, our concordance rate is the highest.

In addition, similar to the findings by Michels et al.,^62^ and by using the same commercial probe, the mean percentage of positive cells by FISH in *RET* rearranged samples in our cohort (55%) was largely above the most often used 15% positivity threshold.

Nevertheless, as shown by our results (discordance rate of 31% between FISH and RNA-seq) and those found in the literature, *RET*-FISH can yield false-positive results.^23^^,^^29^, ^30^, ^31^ Indeed, all rearrangements in the *RET* locus are detected by FISH, independent of whether they result in a transcribed oncogenic fusion or not (in-frame versus out-of-frame rearrangements). As reported in the literature, other reasons for false-positivity using FISH can include statistical sampling effects in borderline samples (percent of rearranged cells close to the positivity threshold) and the presence of multiple copies (gain or amplification) of the target gene.^63^^,^^64^ Nevertheless, in our cohort, out of the 10 false-positive samples (*RET*-FISH positive and RNA-seq negative), only one revealed a borderline positivity (18% of rearranged cells), and it also revealed multiple copies of the *RET* locus. No other RNA-seq negative sample was borderline positive (between 15% and 20% of rearranged nuclei) or presented an augmented number of copies of the *RET* locus. The mean number of positive nuclei in the discordant (FISH false-positive) samples was 47%, which is far away from the positivity threshold. Therefore, the main reason for false positivity we could find in our cohort was the presence of non-transcribed DNA rearrangement of the RET locus. Interestingly, a higher rate of samples revealed isolated 3′ signals in the 10 FISH positive and RNA-seq negative samples compared with the rest of our cohort, with the limit of the relatively low number of cases analyzed. Therefore, when using FISH for the detection of *RET* rearrangements, the use of an orthogonal technique able to detect all transcribed *RET* fusions (regardless of the fusion partners) to confirm all *RET*-FISH positive cases is essential to not treat patients who are not likely to benefit from a *RET*-targeted therapy.

As previously noted, false-negative results by FISH have also been reported in cohorts where samples were tested by both FISH and NGS.^29^^,^^31^^,^^49^^,^^50^^,^^65^ Limited reliability of break-apart FISH because of statistical sampling effects in borderline samples has also been reported as one of the reasons leading to false-positivity using FISH.^63^ A systematic selection of the areas analyzed by FISH should also always be performed by a pathologist to avoid the counting of benign nuclei. Nevertheless, it must be noted that all the cases we found in our literature review that were initially detected using an alternative molecular method (mostly RNA-seq) were found positive by *RET*-FISH, suggesting a good sensitivity of *RET*-FISH.

In view of the limits of the FISH technique for the detection of targetable fusions, RNA-based NGS (RNA-seq) has become the preferred molecular testing option for *RET* fusions, together with other biomarkers, because of its increasingly feasible, multigene testing capability and cost-effectiveness, and has replaced FISH as the technique used for *RET*-rearrangement testing in our laboratory since 2021.

In summary, FISH turn-around time is short (1–2 d) and requires small amounts of tissue, therefore can be used as a screening method for the detection of *RET* rearrangements in the absence of a clinically validated antibody. Nevertheless, FISH-positive and equivocal findings have to be validated by an orthogonal technique, such as RNA-seq, to ensure the detected fusion is indeed a functional oncogenic aberration, to select patients who might benefit from RET-targeted therapy.

## CRediT Authorship Contribution Statement

**AnneMc Leer:** Conceptualization, Methodology, Validation, Investigation, Resources, Data curation, Writing - original draft, Writing - review & editing, Supervision, Project administration.

**Julie Mondet:** Validation, Investigation, Writing - review & editing.

**Nelly Magnat:** Validation, Investigation.

**Mailys Mersch:** Investigation.

**Diane Giovannini:** Investigation, Resources, Writing - review & editing.

**Camille Emprou:** Investigation, Resources, Writing - review & editing.

**Anne-Claire Toffart:** Investigation, Resources, Writing - review & editing.

**Nathalie Sturm:** Investigation, Resources, Writing - review & editing.

**Sylvie Lantuéjoul:** Investigation, Resources, Writing - review & editing.

**David Benito:** Conceptualization, Methodology, Validation, Resources, Data curation, Writing - original draft, Writing - review & editing, Supervision.

## Disclosure

Dr. Mc Leer has declared consulting fees from 10.13039/100015756Janssen-Cilag, Eli Lilly, Takeda, JFR Access, Pfizer, payment for presentations from Amgen, AstraZeneca, Cancerodigest, Edimark, Janssen-Cilag and support for attending meetings from Amgen, AstraZeneca, Janssen-Cilag, Pfizer. Dr. Toffart has declared consulting fees from 10.13039/100004325AstraZeneca, 10.13039/100002491Bristol-Myers Squibb, 10.13039/100002429Amgen, 10.13039/501100014382Ipsen, Janssen, 10.13039/100009947Merck Sharp & Dohme, 10.13039/100004319Pfizer, 10.13039/100004337Roche, Sanofi, Takeda, payment for presentations from AstraZeneca, Bristol-Myers Squibb, Amgen, Ipsen, Janssen, Merck Sharp & Dohme, Pfizer, Roche, Sanofi, Takeda, payment for expert testimony from AstraZeneca, Bristol-Myers Squibb, Amgen, Ipsen, Janssen, Merck Sharp & Dohme, Pfizer, Roche, Sanofi, Takeda and support for attending meetings from AstraZeneca, Pfizer, Roche, Takeda. Dr. Benito is a full-time employee at Eli Lilly Co. The remaining authors declare no conflict of interest.
