# Supplementary material for: Rearranged During Transfection Rearrangement Detection by Fluorescence In Situ Hybridization Compared With Other Techniques in NSCLC
Source: JTO Clin Res Rep. 2024 Aug 29;5(12):100714. doi: 10.1016/j.jtocrr.2024.100714 (PMC11539407; doi:10.1016/j.jtocrr.2024.100714)
Supplement: Supplementary Material [file mmc1.pdf]

Supplementary Table S1: Complete list of studies published between 2000 and 2022 mentioning *RET* FISH in their methodology (in bold, studies reported in Tables 1a and 1b).

2000	Cinti	RET rearrangements in papillary thyroid carcinomas and adenomas detected by interphase FISH	https://pubmed.ncbi.nlm.nih.gov/10773666/
2000	Corvi	RET/PCM-1: a novel fusion gene in papillary thyroid carcinoma	https://pubmed.ncbi.nlm.nih.gov/10980597/
2001	Corvi	Frequent RET rearrangements in thyroid papillary microcarcinoma detected by interphase fluorescence in situ hybridization	https://pubmed.ncbi.nlm.nih.gov/11742034/
2001	Corvi	RET rearrangements in familial papillary thyroid carcinomas	https://pubmed.ncbi.nlm.nih.gov/11463498/
2006	Zhu	Prevalence of RET/PTC rearrangements in thyroid papillary carcinomas: effects of the detection methods and genetic heterogeneity	https://pubmed.ncbi.nlm.nih.gov/16772343/
**2012**	**Takeuchi**	**RET, ROS1 and ALK fusions in lung cancer**	**https://pubmed.ncbi.nlm.nih.gov/22327623/**
**2012**	**Kohno**	**KIF5B-RET fusions in lung adenocarcinoma**	**https://pubmed.ncbi.nlm.nih.gov/22327624/**
**2012**	**Wang**	**RET fusions define a unique molecular and clinicopathologic subtype of non-small-cell lung cancer**	**https://pubmed.ncbi.nlm.nih.gov/23150706/**
**2012**	**Suehara**	**Identification of KIF5B-RET and GOPC-ROS1 fusions in lung adenocarcinomas through a comprehensive mRNA-based screen for tyrosine kinase fusions**	**https://pubmed.ncbi.nlm.nih.gov/23052255/**
**2012**	**Sasaki**	**RET expression and detection of KIF5B/RET gene rearrangements in Japanese lung cancer**	**https://pubmed.ncbi.nlm.nih.gov/23342255/**
2013	Drilon	Response to Cabozantinib in patients with RET fusion-positive lung adenocarcinomas	https://pubmed.ncbi.nlm.nih.gov/23533264/
2013	Caria	Assessing RET/PTC in thyroid nodule fine-needle aspirates: the FISH point of view	https://pubmed.ncbi.nlm.nih.gov/23722226/
**2013**	**Go**	**Diagnostic method for the detection of KIF5B-RET transformation in lung adenocarcinoma**	**https://pubmed.ncbi.nlm.nih.gov/23932363/**
**2013**	**Borrelli**	**KIF5B/RET fusion gene analysis in a selected series of cytological specimens of EGFR, KRAS and EML4-ALK wild-type adenocarcinomas of the lung**	**https://pubmed.ncbi.nlm.nih.gov/23891510/**
**2014**	**Tsuta**	**RET-rearranged non-small-cell lung carcinoma: a clinicopathological and molecular analysis**	**https://pubmed.ncbi.nlm.nih.gov/24504365/**
**2014**	**Pan**	**ALK, ROS1 and RET fusions in 1139 lung adenocarcinomas: a comprehensive study of common and fusion pattern-specific clinicopathologic, histologic and cytologic features**	**https://pubmed.ncbi.nlm.nih.gov/24629636/**
**2014**	**Lira**	**A single-tube multiplexed assay for detecting ALK, ROS1, and RET fusions in lung cancer**	**https://pubmed.ncbi.nlm.nih.gov/24418728/**
2014	Mizukami	Molecular mechanisms underlying oncogenic RET fusion in lung adenocarcinoma	https://pubmed.ncbi.nlm.nih.gov/24722152/
2015	Zhang	An Evaluation and Recommendation of the Optimal Methodologies to Detect RET Gene Rearrangements in Papillary Thyroid Carcinoma	https://pubmed.ncbi.nlm.nih.gov/25407564/
**2015**	**Lee**	**Comprehensive analysis of RET and ROS1 rearrangement in lung adenocarcinoma**	**https://pubmed.ncbi.nlm.nih.gov/25234288/**

2015	Colato	Break-apart interphase fluorescence in situ hybridization assay in papillary thyroid carcinoma: on the road to optimizing the cut-off level for RET/PTC rearrangements	https://pubmed.ncbi.nlm.nih.gov/25698220/
**2015**	**Kim**	**KIF5B-RET Fusion gene may coincide oncogenic mutations of EGFR or KRAS gene in lung adenocarcinomas**	**https://pubmed.ncbi.nlm.nih.gov/26268359/**
2015	Platt	A retrospective analysis of RET translocation, gene copy number gain and expression in NSCLC patients treated with vandetanib in four randomized Phase III studies	https://pubmed.ncbi.nlm.nih.gov/25881079/
2015	Subbiah	Systemic and CNS activity of the RET inhibitor vandetanib combined with the mTOR inhibitor everolimus in KIF5B-RET re-arranged non-small cell lung cancer with brain metastases	https://pubmed.ncbi.nlm.nih.gov/25982012/
2015	Grubbs	RET Fusion as a Novel Driver of Medullary Thyroid Carcinoma	https://pubmed.ncbi.nlm.nih.gov/25546157/
2015	Drilon	Broad, Hybrid Capture-Based Next-Generation Sequencing Identifies Actionable Genomic Alterations in Lung Adenocarcinomas Otherwise Negative for Such Alterations by Other Genomic Testing Approaches	https://pubmed.ncbi.nlm.nih.gov/25567908/
2016	Michels	Clinicopathological Characteristics of RET Rearranged Lung Cancer in European Patients	https://pubmed.ncbi.nlm.nih.gov/26762747/
2016	Lee	Identification of a novel partner gene, KIAA1217, fused to RET: Functional characterization and inhibitor sensitivity of two isoforms in lung adenocarcinoma	https://pubmed.ncbi.nlm.nih.gov/27150058/
2016	Lu	1p/19q codeletion and RET rearrangements in small-cell lung cancer	https://pubmed.ncbi.nlm.nih.gov/27366094/
2016	Drilon	Cabozantinib in patients with advanced RET-rearranged non-small-cell lung cancer: an open-label, single-centre, phase 2, single-arm trial	https://pubmed.ncbi.nlm.nih.gov/27825636/
2016	Catelain	Detection of Gene Rearrangements in Circulating Tumor Cells: Examples of ALK-, ROS1-, RET-Rearrangements in Non-Small-Cell Lung Cancer and ERG-Rearrangements in Prostate Cancer	https://pubmed.ncbi.nlm.nih.gov/28560674/
**2016**	**Song**	**Clinicopathological characteristics and survival of ALK, ROS1 and RET rearrangements in non-adenocarcinoma non-small cell lung cancer patients**	**https://pubmed.ncbi.nlm.nih.gov/27635639/**
2017	Lee	Vandetanib in pretreated patients with advanced non-small cell lung cancer-harboring RET rearrangement: a phase II clinical trial	https://pubmed.ncbi.nlm.nih.gov/27803005/
2017	Dugay	Clinicopathological characteristics of ROS1- and RET- rearranged NSCLC in caucasian patients: Data from a cohort of 713 non-squamous NSCLC lacking KRAS/EGFR/HER2/BRAF/PIK3CA/ALK alterations	https://pubmed.ncbi.nlm.nih.gov/28881815/
**2017**	**Yoh**	**Vandetanib in patients with previously treated RET-rearranged advanced non-small-cell lung cancer (LURET): an open-label, multicentre phase 2 trial**	**https://pubmed.ncbi.nlm.nih.gov/27825616/**
**2017**	**Song**	**Clinicopathologic characteristics, genetic variability and therapeutic options of RET rearrangements patients in lung adenocarcinoma**	**https://pubmed.ncbi.nlm.nih.gov/27794403/**
**2017**	**Reguart**	**Identification of ALK, ROS1, and RET Fusions by a Multiplexed mRNA-Based Assay in Formalin-Fixed, Paraffin-Embedded Samples from Advanced Non-Small-Cell Lung Cancer Patients**	**https://pubmed.ncbi.nlm.nih.gov/28073897/**

**2017**	**Rogers**	**Multiplexed transcriptome analysis to detect ALK, ROS1 and RET rearrangements in lung cancer**	**https://pubmed.ncbi.nlm.nih.gov/28181564/**
2017	Zhang	Identification of a novel KIF13A-RET fusion in lung adenocarcinoma by next-generation sequencing	https://pubmed.ncbi.nlm.nih.gov/29571998/
2017	Uguen	Asbestos-related lung cancers: A retrospective clinical and pathological study	https://pubmed.ncbi.nlm.nih.gov/28685091/
**2017**	**Tanaka**	**Unique prevalence of oncogenic genetic alterations in young patients with lung adenocarcinoma**	**https://pubmed.ncbi.nlm.nih.gov/28177518/**
2017	Tang	Coexistent genetic alterations involving ALK, RET, ROS1 or MET in 15 cases of lung adenocarcinoma	https://pubmed.ncbi.nlm.nih.gov/28914263/
2018	Skalova	Molecular Profiling of Mammary Analog Secretory Carcinoma Revealed a Subset of Tumors Harboring a Novel ETV6-RET Translocation: Report of 10 Cases	https://pubmed.ncbi.nlm.nih.gov/29076873/
**2018**	**Piton**	**Ligation-dependent RT-PCR: a new specific and low-cost technique to detect ALK, ROS, and RET rearrangements in lung adenocarcinoma**	**https://pubmed.ncbi.nlm.nih.gov/29251734/**
2018	Uguen	Asbestos-related lung cancers are rarely associated with ALK, ROS1 and RET rearrangements	https://pubmed.ncbi.nlm.nih.gov/29519903/
**2018**	**Kim**	**Detection of RET (rearranged during transfection) variants and their downstream signal molecules in RET rearranged lung adenocarcinoma patients**	**https://pubmed.ncbi.nlm.nih.gov/29549897/**
2018	Kim	NCOA4-RET fusion in colorectal cancer: Therapeutic challenge using patient-derived tumor cell lines	https://pubmed.ncbi.nlm.nih.gov/30210625/
2018	Andreasen	The ETV6-RET Gene Fusion Is Found in ETV6-rearranged Low-grade Sinonasal Adenocarcinoma Without NTRK3 Involvement	https://pubmed.ncbi.nlm.nih.gov/29683817/
2018	Weinreb	Recurrent RET Gene Rearrangements in Intraductal Carcinomas of Salivary Gland	https://pubmed.ncbi.nlm.nih.gov/29443014/
2018	Salvi	Evaluation of RET Gene Rearrangement by Fluorescence In Situ Hybridization in Malignant Mesothelioma	https://pubmed.ncbi.nlm.nih.gov/29258668/
2018	Ferrara	Clinical and Translational Implications of RET Rearrangements in Non-Small Cell Lung Cancer	https://pubmed.ncbi.nlm.nih.gov/29128428/
2018	Pietrantonio	RET fusions in a small subset of advanced colorectal cancers at risk of being neglected	https://pubmed.ncbi.nlm.nih.gov/29538669/
2018	Skalova	Molecular profiling of salivary gland intraductal carcinoma revealed a subset of tumors harboring NCOA4-RET and novel TRIM27-RET fusions: a report of 17 cases	https://pubmed.ncbi.nlm.nih.gov/30045065/
2018	Offin	Immunophenotype and Response to Immunotherapy of RET-Rearranged Lung Cancers	https://pubmed.ncbi.nlm.nih.gov/31192313/
**2018**	**Velizheva**	**Targeted next-generation-sequencing for reliable detection of targetable rearrangements in lung adenocarcinoma-a single center retrospective study**	**https://pubmed.ncbi.nlm.nih.gov/29580750/**
2019	Musholt	Detection of RET rearrangements in papillary thyroid carcinoma using RT-PCR and FISH techniques - A molecular and clinical analysis	https://pubmed.ncbi.nlm.nih.gov/30472213/
2019	Michal	S100 and CD34 positive spindle cell tumor with prominent perivascular hyalinization and a novel NCOA4-RET fusion	https://pubmed.ncbi.nlm.nih.gov/30938880/

**2019**	**Shang**	**Histology and oncogenic driver alterations of lung adenocarcinoma in Chinese**	**https://pubmed.ncbi.nlm.nih.gov/31285953/**
2019	Sakai	Performance of Oncomine Fusion Transcript kit for formalin-fixed, paraffin-embedded lung cancer specimens	https://pubmed.ncbi.nlm.nih.gov/30972901/
2019	Staubitz	Evaluation of RET Gene Rearrangement by Fluorescence In Situ Hybridization in Malignant Mesothelioma	https://pubmed.ncbi.nlm.nih.gov/31425920/
2019	Skalova	NCOA4-RET and TRIM27-RET Are Characteristic Gene Fusions in Salivary Intraductal Carcinoma, Including Invasive and Metastatic Tumors: Is "Intraductal" Correct?	https://pubmed.ncbi.nlm.nih.gov/31162284/
2019	Prager	Results of the extended analysis for cancer treatment (EXACT) trial: a prospective translational study evaluating individualized treatment regimens in oncology	https://pubmed.ncbi.nlm.nih.gov/30847023/
2019	Lee	Characteristics and outcomes of RET-rearranged Korean non-small cell lung cancer patients in real-world practice	https://pubmed.ncbi.nlm.nih.gov/32083304/
**2020**	**Chen**	**Identifying a wide range of actionable variants using capture-based ultra-deep targeted sequencing in treatment-naive patients with primary lung adenocarcinoma**	**https://pubmed.ncbi.nlm.nih.gov/32269691/**
2020	Lu	Intraductal Carcinoma of Salivary Glands Harboring TRIM27-RET Fusion with Mixed Low Grade and Apocrine Types	https://pubmed.ncbi.nlm.nih.gov/30610524/
2020	Chou	RET gene rearrangements occur in a subset of pancreatic acinar cell carcinomas	https://pubmed.ncbi.nlm.nih.gov/31558784/
**2020**	**Tan**	**Molecular Characterization and Clinical Outcomes in RET-Rearranged NSCLC**	**https://pubmed.ncbi.nlm.nih.gov/32866654/**
2020	Liu	Pitfalls in RET fusion detection using break-apart FISH probes in papillary thyroid carcinoma	https://pubmed.ncbi.nlm.nih.gov/33382428/
2020	Cheek	Uterine inflammatory myofibroblastic tumors in pregnant women with and without involvement of the placenta: a study of 6 cases with identification of a novel TIMP3-RET fusion	https://pubmed.ncbi.nlm.nih.gov/31917155/
2020	Loong	Novel TFG-RET fusion in a spindle cell tumour with S100 and CD34 coexpresssion	https://pubmed.ncbi.nlm.nih.gov/31411754/
2020	Sussman	Validation of a Next-Generation Sequencing Assay Targeting RNA for the Multiplexed Detection of Fusion Transcripts and Oncogenic Isoforms	https://pubmed.ncbi.nlm.nih.gov/31211614/
2020	Rooper	Salivary Intraductal Carcinoma Arising within Intraparotid Lymph Node: A Report of 4 Cases with Identification of a Novel STRN-ALK Fusion	https://pubmed.ncbi.nlm.nih.gov/32661669/
2020	Bishop	Oncocytic Intraductal Carcinoma of Salivary Glands: A Distinct Variant with TRIM33-RET Fusions and BRAF V600E mutations	https://pubmed.ncbi.nlm.nih.gov/33135196/
**2020**	**Sokolova**	**Multiplex fast FISH assay for detecting ROS1, RET and MET aberrations in FFPE specimens using BioView image analysis**	**https://cancerres.aacrjournals.org/content/80/16_Supplement/4256.short**
2020	Skalova	Expanding the Molecular Spectrum of Secretory Carcinoma of Salivary Glands With a Novel VIM-RET Fusion	https://pubmed.ncbi.nlm.nih.gov/32675658/
**2021**	**Baker**	**Analytical Accuracy of RET Fusion Detection by Break-Apart Fluorescence In Situ Hybridization**	**https://pubmed.ncbi.nlm.nih.gov/34232984/**
**2021**	**Yang**	**A Performance Comparison of Commonly Used Assays to Detect RET Fusions**	**https://pubmed.ncbi.nlm.nih.gov/33272981/**

2021	Yamamoto	Pan-Trk immunoreactivity, ETV6-NTRK3 fusion subtypes and RET rearrangement in salivary secretory carcinoma	https://pubmed.ncbi.nlm.nih.gov/33301751/
**2021**	**Takeuchi**	**Phase 1/2 study of alectinib in RET-rearranged previously-treated non-small cell lung cancer (ALL-RET)**	**https://tlcr.amegroups.com/article/view/46077/html**
2021	Campanha Novaes	Simultaneous analysis of ALK, RET, and ROS1 gene fusions by NanoString in Brazilian lung adenocarcinoma patients	https://tlcr.amegroups.com/article/view/48020/html
**2021**	**Radonic**	**RET FISH analysis is a sensitive but highly unspecific screening method for RET fusions in lung cancer**	**https://pubmed.ncbi.nlm.nih.gov/33588111/**
**2022**	**Feng**	**Clinicopathologic characteristics and diagnostic methods of RET rearrangement in Chinese non-small cell lung cancer patients**	**https://pubmed.ncbi.nlm.nih.gov/35529790/**
2022	Shi	Identifification of a novel LDLR-RET Fusion in Lung Adenocarcinoma	https://pubmed.ncbi.nlm.nih.gov/35524867/
**2022**	**Ambrosini-Spaltro**	**The role of next-generation sequencing in detecting gene fusions with known and unknown partners: a single-center experience with methodologies' integration**	**https://pubmed.ncbi.nlm.nih.gov/35181377/**
2022	Liu	Profiling of gene fusion involving targetable genes in Chinese gastric cancer	https://pubmed.ncbi.nlm.nih.gov/36160735/
2023	Chen	Highly sensitive droplet digital PCR for detection of RET fusion in papillary thyroid cancer	https://pubmed.ncbi.nlm.nih.gov/37081420/

Supplementary Table S2. ***RET*** **FISH studies on lung cancer reported in the literature.**

Author	Year	LUNG cases studied for RET	RET cases detected (any technique)	techniques	RET FISH+ cases	RET FISH+ cases compare with other molecular technique	RET+ FISH samples confirmed by other molecular techinique	probe mentioned	cutoff	pattern (positive)	comments
Suehara[21]	2012	69	1	Nanostring, FISH	1	1	1	BAC probe	10%	split signals	FISH for confirmation
Kohno[45]	2012	319	6	RTqPCR, Sanger, FISH	6	6	6	GSP Research Inc.	NA	NA	FISH for confirmation
Sasaki[10]	2012	157	3	RT-PCR, IHC, FISH	*2*	*2*	*2*	KIF5B/RET (GSP Research Inc.)	15%	KIF5B/RET *	*Not a break apart RET probe*
Wang[14]	2012	936	13	RT-PCR, IHC, FISH	13	13	13	*NA*	> 20%	split signals	FISH for confirmation
Takeuchi[1]	2012	1529	23	RTqPCR, FISH	22	22	12	*NA*	NA	NA	screening by FISH
Go[8]	2013	53	3	NGS (transcriptome), FISH	3	3	3	Macrogen	> 15%	split signals	screening by FISH
Drilon[48]	2013	31	5	FISH , RT-PCR	5	0	0	NA	10%	split signals or isolated 3’	*No technique comparison*
Borrelli[15]	2013	49	1	RTqPCR, FISH	1	1	1	Leica	15%	split signals or isolated 5’ signals	FISH for confirmation
Pan[16]	2014	1139	15	RTqPCR, FISH	15	15	15	BAC probe	>15%	NA	FISH for confirmation
Mizukami[43]	2014	352	10	RT-PCR, FISH only in 3 samples	*2*	*NA*	*NA*	CSL	NA	NA	*Not clear validation*
Tsuta[2]	2014	1874	50	IHC, RTqPCR, FISH	50	29	16	CSL	⩾20%	split signals or isolated 3’	screening by FISH

Lira[22]	2014	295	15	Nanostring, FISH	15	15	15	Zytovision	NA	NA	FISH for confirmation
Platt[47]	2015	944	7	IHC,FISH	NA	NA	NA	BAC probe	>10%	split signals	*no validation by another molecular technique*
Subbiah[41]	2015	1	case report	NGS (exome), FISH	1	1	0	CymoGen	NA	NA	*case report*
Drilon[49]	2015	31	3	NGS-DNA, FISH	0	*NA*	*NA*	NA	NA	NA	*Incomplete comparison data*
Lee[13]	2015	295	15	IHC, FISH, NanoString	14	14	14	Zytovision	> 15 %	split signals	FISH for confirmation
Kim[17]	2015	154	9	RTqPCR, FISH	9	9	9	Zytovision	> 15 %	split signals	FISH for confirmation
Drilon	*2016*	*NA*	*26*	*FISH, NGS*	*NA*	*NA*	*NA*	*BAC probe*	*NA*	*NA*	*No technique comparison*
Song[12]	2016	615	12	RTqPCR, FISH, NGS-RNA	11	11	11	Abbott	>15%	split signals	FISH for confirmation
Lee[36]	2016	1	1	IHC, RET	1	1	1	Zytovision	NA	NA	*case report*
Michels[35]	2016	997	22	FISH	22	NA	NA	Zytovision	≥20% and ≥15%	split signals or isolated 3’	*No comparison. Only FISH was used*
Lu[34]	2016	32	0	FISH	0	0	0	Zytovision	>15%	split signals	*No comparison. None of the specimens had a RET rearrangement*
Pailler[31]	2016	NA	NA	FISH, FCM	NA	NA	NA	Zytovision	NA	NA	*No validation by molecular technique*
Tanaka[20]	2017	46	4	rtPCR, FISH	4	4	2	Agilent	NA	NA	FISH for confirmation
Lee[44]	2017	306	26	FISH, IHC, PCR, NGS-DNA	26	10	8	Macrogen	NA	NA	screening by FISH
Yoh[50]	2017	1433	34	RTqPCR, FISH	19	19	19	*NA*	> 15%	NA	FISH for confirmation
Zhang[42]	2017	1	case report	NGS-DNA, FISH	1	1	1	CytoTest	NA	NA	case report
Song[18]	2017	385	2	RTqPCR, FISH	2	2	2	Abbott	>15%	split signals	FISH for confirmation
Reguart[19]	2017	98	2	IHC, RT-PCR, Nanostring, FISH	2	2	2	Zytovision	15%	split signals and/or single 3'	FISH for confirmation
Dugay[30]	2017	713	18	FISH	18	NA	NA	Zytovision	15%	split signals or isolated 3’	*No comparison. Only FISH was used*
Catelain[32]	2017	5	5	FISH, FCM	NA	NA	NA	Zytovision	NA	NA	*No validation by molecular technique*
Rogers[3]	2017	51	1	Nanostring, FISH	1	1	1 (only by Agena)	Zytovision	≥ 15%	split signals	screening by FISH
Tang[40]	2018	2379	33	FISH, NGS-DNA	33	NA	NA	CymoGen	>3.1%, >4.4% >7.9%	NA	*No technique comparison*
Uguen[38]	2018	56	1	IHC, FISH	1	NA	NA	Leica	NA	NA	*No technique comparison. 1 case was detected only by FISH.*

Velizheva[23]	2018	52	1	NGS-RNA, FISH	1	1	1	Zytovision	≥ 15%	split signals	FISH for confirmation
Piton[9]	2018	39	1	Ligation-dependent RT-PCR, FISH	1	1	1	Zytovision	< 20%	split signals or isolated 3'	screening by FISH
Kim[7]	2018	581	51	FISH,IHC, RT-PCR, Nanostring	51	51	3	Zytovision	NA	split signals	screening by FISH
Shang[24]	2019	897	20	RT-PCR, FISH	20	20	20	BAC probe	NA	NA	FISH for confirmation
Offin[46]	2019	74	74	NGS-DNA, RT-PCR (RNA), FISH	11	NA	NA	Metasystems	NA	NA	*No technique comparison*
Prager[39]	2019	NA	0	NGS-DNA, IHC, FISH	NA	NA	NA	Leica	NA	NA	*No validation between techniques. Data not shown*
Lee[51]	2020	59	59	NGS-RNA, FISH	8	*NA*	*NA*	*NA*	NA	NA	*Not clear validation*
Chen[24]	2020	372	10	NGS-DNA, FISH	10	10	10	CytoTest	NA	NA	FISH for confirmation
Sokolova[37]	2020	55	3	NGS-RNA or RT-PCR, FISH	3	3	3	Abbott	>15%	NA	FISH for confirmation
Tan[4]	2020	64	30	NGS-RNA, FISH	30	9	6	Abbott	≥ 15%	split signals or isolated 3'	screening by FISH (3FP and 2FN)
Takeuchi[11]	2021	4,552	119	NGS-RNA, RT-PCR, FISH	34	34	34	*NA*	NA	NA	FISH for confirmation
Baker[6]	2021	21	8	NGS-RNA, FISH	8	8	5	Abbott	13% and 19%	split signals or isolated 3' or isolated 5'	screening by FISH
Radonic[5]	2021	2858	48	NGS-RNA, FISH	48	30	9	Abbott, Agilent, Leica, Cytocell	≥ 15%	split signals or isolated 3'	screening by FISH (21FP)
Yang[26]	2021	5920	99	NGS-DNA,FISH, IHC	27	27	27	Zytovision	≥10%	split signals or isolated 3'	FISH for confirmation
Campanha Novaes[33]	2021	134	2	Nanostring, FISH	0	0	0	Zytovision	> 15%	NA	*Inconclusive results*
Shi[27]	2022	1	1	NGS-DNA, FISH	1	1	1	LBP	≥15%	split signals or isolated 3'	*case report*
Feng[28]	2022	9,101	138	NGS-DNA, NGS-RNA, FISH, IHC	25	25	25	LBP	≥15%	split signals or isolated 3'	FISH for confirmation
Ambrosini-Spaltro[29]	2022	1174	21	RNA-NGS, FISH	2	2	2	Abbott	10%	NA	FISH for confirmation

NA: Not available
Zytovision: ZytoLight SPEC RET Dual Break Apart Probe (Zytovision GmbH, Bremerhaven, Germany)
Abbott: Vysis RET break-apart (Abbott Molecular, Abbott Park, Illinois)
Leica: RET (10q11) Kreatech (Leica Biosystems, Wetzlar, Germany)
Agilent: Dako SureFISH RET BA (Agilent, Santa Clara, California)
CymoGen: Clear-View FISH RET dual color, breakapart probe from CymoGen DX (Biocare Medical)
CytoTest: RET break apart FISH probe (CytoTest, USA)
CSL: RET dual-colour break-apart probe (Chromosome Science Labo, Inc., Sapporo, Japan)
Macrogen: (LSI) RET dual-color break-apart probe (Macrogen Inc., Seoul, Korea)
GSP: break-apart probe for RET (GSP laboratory, Kawasaki, Japan).
LBP: RET (10q11) dual-color break-apart rearrangement probe (LBP Medicine Science and Technology, Guangzhou)
Metasystems: 10q11 and 6q22 break apart probe (Metasystems, Altussheim, Germany)

Cytocell: Cytocell Aquarius (Cytocell Cambridge, UK)
*one probe was a KIF5B/RET probe developed at GSP Research Inc.

Supplementary Table S3: Commercial break-apart RET FISH probes used in the literature

Manufacturer	Commercial break-apart *RET* FISH probes mentioned in a total of 40 publications
ZytoVision[3,7,9,13,17,19,22,23,26,30–36]	16 (37%)
Abbott[4–6,12,18,29,37]	7 (16%)
Leica[5,15,38,39]	4 (9%)
Agilent[5,20]	2 (5%)
CymoGen Dx[40,41]	2 (5%)
CytoTest[25,42]	2 (5%)
CSL[2,43]	2 (5%)
Macrogen[8,44]	2 (5%)
GSP Research Inc.[27,45]	1+1* (5%)
LBP[28]	2 (5%)
Metasystems[46]	1 (2%)
Cytocell[5]	1 (2%)

Zytovision: ZytoLight SPEC RET Dual Break Apart Probe (Zytovision GmbH, Bremerhaven, Germany)
Abbott: Vysis RET break-apart (Abbott Molecular, Abbott Park, Illinois)
Leica: RET (10q11) Kreatech (Leica Biosystems, Wetzlar, Germany)
Agilent: Dako SureFISH RET BA (Agilent, Santa Clara, California)
CymoGen: Clear-View FISH RET dual color, breakapart probe from CymoGen DX (Biocare Medical)
CytoTest: RET break apart FISH probe (CytoTest, USA)
CSL: RET dual-colour break-apart probe (Chromosome Science Labo, Inc., Sapporo, Japan)
Macrogen: (LSI) RET dual-color break-apart probe (Macrogen Inc., Seoul, Korea)
GSP: break-apart probe for RET (GSP laboratory, Kawasaki, Japan).

LBP: RET (10q11) dual-color break-apart rearrangement probe (LBP Medicine Science and Technology, Guangzhou)
Metasystems: 10q11 and 6q22 break apart probe (Metasystems, Altussheim, Germany)
Cytocell: Cytocell Aquarius (Cytocell Cambridge, UK)
*one probe was a KIF5B/RET probe developed at GSP Research Inc.

Supplementary Table S4: RET-FISH patterns

RET-FISH patterns considered to be positive	Number of publications
split signals and/or isolated 3’ signals	11[2,4,5,9,19,26-28,30,35,48]
split signals only	12 [3,17,23,34,36,38,48]
split signals and/or isolated 5’ signals	1[19]
KIF5B-RET fusion	1[8]
split signals or isolated 3’ signals/isolated 5’ signals	1[6]
**Total**	**26**

References used in Tables S2, S3 and S4

1. Takeuchi K, Soda M, Togashi Y, et al. RET, ROS1 and ALK fusions in lung cancer. *Nat Med*. 2012;18(3):378-381. doi:10.1038/nm.2658

2. Tsuta K, Kohno T, Yoshida A, et al. RET-rearranged non-small-cell lung carcinoma: a clinicopathological and molecular analysis. *Br J Cancer*. 2014;110(6):1571-1578. doi:10.1038/bjc.2014.36

3. Rogers TM, Arnau GM, Ryland GL, et al. Multiplexed transcriptome analysis to detect ALK, ROS1 and RET rearrangements in lung cancer. *Sci Rep*. 2017;7(1):42259. doi:10.1038/srep42259

4. Tan AC, Seet AOL, Lai GGY, et al. Molecular Characterization and Clinical Outcomes in RET-Rearranged NSCLC. *J Thorac Oncol*. 2020;15(12):1928-1934. doi:10.1016/j.jtho.2020.08.011

5. Radonic T, Geurts-Giele WRR, Samsom KG, et al. RET Fluorescence In Situ Hybridization Analysis Is a Sensitive but Highly Unspecific Screening Method for RET Fusions in Lung Cancer. *J Thorac Oncol*. 2021;16(5):798-806. doi:10.1016/j.jtho.2021.01.1619

6. Baker JA, Sireci AN, Marella N, et al. Analytical Accuracy of RET Fusion Detection by Break-Apart Fluorescence In Situ Hybridization. *Arch Pathol Lab Med*. Published online July 8, 2021. doi:10.5858/arpa.2020-0376-OA

7. Kim JO, Shin JY, Kim MY, et al. Detection of RET (rearranged during transfection) variants and their downstream signal molecules in RET rearranged lung adenocarcinoma patients. *Surg Oncol*. 2018;27(1):106-113. doi:10.1016/j.suronc.2018.01.006

8. Go H, Jung YJ, Kang HW, et al. Diagnostic method for the detection of KIF5B-RET transformation in lung adenocarcinoma. *Lung Cancer*. 2013;82(1):44-50. doi:10.1016/j.lungcan.2013.07.009

9. Piton N, Ruminy P, Gravet C, et al. Ligation-dependent RT-PCR: a new specific and low-cost technique to detect ALK, ROS, and RET rearrangements in lung adenocarcinoma. *Lab Invest*. 2018;98(3):371-379. doi:10.1038/labinvest.2017.124

10. Sasaki H, Shimizu S, Tani Y, et al. RET expression and detection of *KIF5B* / *RET* gene rearrangements in Japanese lung cancer. *Cancer Med*. 2012;1(1):68-75. doi:10.1002/cam4.13

11. Takeuchi S, Yanagitani N, Seto T, et al. Phase 1/2 study of alectinib in RET-rearranged previously-treated non-small cell lung cancer (ALL-RET). *Transl Lung Cancer Res*. 2021;10(1):314-325. doi:10.21037/tlcr-20-549

12. Song Z, Yu X, Zhang Y. Clinicopathologic characteristics, genetic variability and therapeutic options of RET rearrangements patients in lung adenocarcinoma. *Lung Cancer*. 2016;101:16-21. doi:10.1016/j.lungcan.2016.09.002

13. Lee SE, Lee B, Hong M, et al. Comprehensive analysis of RET and ROS1 rearrangement in lung adenocarcinoma. *Mod Pathol*. 2015;28(4):468-479. doi:10.1038/modpathol.2014.107

14. Wang R, Hu H, Pan Y, et al. *RET* Fusions Define a Unique Molecular and Clinicopathologic Subtype of Non–Small-Cell Lung Cancer. *J Clin Oncol*. 2012;30(35):4352-4359. doi:10.1200/JCO.2012.44.1477

15. Borrelli N, Giannini R, Proietti A, et al. KIF5B/RET fusion gene analysis in a selected series of cytological specimens of EGFR, KRAS and EML4-ALK wild-type adenocarcinomas of the lung. *Lung Cancer*. 2013;81(3):377-381. doi:10.1016/j.lungcan.2013.06.026

16. Pan Y, Zhang Y, Li Y, et al. ALK, ROS1 and RET fusions in 1139 lung adenocarcinomas: A comprehensive study of common and fusion pattern-specific clinicopathologic, histologic and cytologic features. *Lung Cancer*. 2014;84(2):121-126. doi:10.1016/j.lungcan.2014.02.007

17. Kim JO, Lee J, Shin JY, et al. KIF5B-RET Fusion gene may coincide oncogenic mutations of EGFR or KRAS gene in lung adenocarcinomas. *Diagn Pathol*. 2015;10(1):143. doi:10.1186/s13000-015-0368-z

18. Song Z, Yu X, Zhang Y. Clinicopathological characteristics and survival of ALK, ROS1 and RET rearrangements in non-adenocarcinoma non-small cell lung cancer patients. *Cancer Biol Ther*. 2017;18(11):883-887. doi:10.1080/15384047.2016.1235660

19. Reguart N, Teixidó C, Giménez-Capitán A, et al. Identification of ALK, ROS1, and RET Fusions by a Multiplexed mRNA-Based Assay in Formalin-Fixed, Paraffin-Embedded Samples from Advanced Non–Small-Cell Lung Cancer Patients. *Clin Chem*. 2017;63(3):751-760. doi:10.1373/clinchem.2016.265314

20. Tanaka K, Hida T, Oya Y, et al. Unique prevalence of oncogenic genetic alterations in young patients with lung adenocarcinoma: Young Patients With Lung Adenocarcinoma. *Cancer*. 2017;123(10):1731-1740. doi:10.1002/cncr.30539

21. Suehara Y, Arcila M, Wang L, et al. Identification of *KIF5B-RET* and *GOPC-ROS1* Fusions in Lung Adenocarcinomas through a Comprehensive mRNA-Based Screen for Tyrosine Kinase Fusions. *Clin Cancer Res*. 2012;18(24):6599-6608. doi:10.1158/1078-0432.CCR-12-0838

22. Lira ME, Choi YL, Lim SM, et al. A Single-Tube Multiplexed Assay for Detecting ALK, ROS1, and RET Fusions in Lung Cancer. *J Mol Diagn*. 2014;16(2):229-243. doi:10.1016/j.jmoldx.2013.11.007

23. Velizheva NP, Rechsteiner MP, Valtcheva N, et al. Targeted next-generation-sequencing for reliable detection of targetable rearrangements in lung adenocarcinoma—a single center retrospective study. *Pathol - Res Pract*. 2018;214(4):572-578. doi:10.1016/j.prp.2018.02.001

24. Shang G, Jin Y, Zheng Q, et al. Histology and oncogenic driver alterations of lung adenocarcinoma in Chinese. *Am J Cancer Res*. 2019;9(6):1212-1223.

25. Chen L, Chen M, Lin J, et al. Identifying a wide range of actionable variants using capture-based ultra-deep targeted sequencing in treatment-naive patients with primary lung adenocarcinoma. *Int J Clin Exp Pathol*. 2020;13(3):525-535.

26. Yang SR, Aypar U, Rosen EY, et al. A Performance Comparison of Commonly Used Assays to Detect RET Fusions. *Clin Cancer Res*. 2021;27(5):1316-1328. doi:10.1158/1078-0432.CCR-20-3208

27. Shi G, Luo Y, Yu Z, Wang Y, Zhu B. Identifification of a novel LDLR-RET Fusion in Lung Adenocarcinoma. *Invest New Drugs*. 2022;40(4):858-860. doi:10.1007/s10637-022-01246-2

28. Feng J, Li Y, Wei B, et al. Clinicopathologic characteristics and diagnostic methods of RET rearrangement in Chinese non-small cell lung cancer patients. *Transl Lung Cancer Res*. 2022;11(4):617-631. doi:10.21037/tlcr-22-202

29. Ambrosini-Spaltro A, Farnedi A, Calistri D, et al. The role of next-generation sequencing in detecting gene fusions with known and unknown partners: a single-center experience with methodologies' integration. *Hum Pathol*. 2022;123:20-30. doi:10.1016/j.humpath.2022.02.005

30. Dugay F, Llamas-Gutierrez F, Gournay M, et al. Clinicopathological characteristics of *ROS1*- and *RET*- rearranged NSCLC in caucasian patients: Data from a cohort of 713 non-squamous NSCLC lacking KRAS/EGFR/HER2/BRAF/PIK3CA/ALK alterations. *Oncotarget*. 2017;8(32):53336-53351. doi:10.18632/oncotarget.18408

31. Pailler E, Oulhen M, Billiot F, et al. Method for semi-automated microscopy of filtration-enriched circulating tumor cells. *BMC Cancer*. 2016;16(1):477. doi:10.1186/s12885-016-2461-4

32. Catelain C, Pailler E, Oulhen M, Faugeroux V, Pommier AL, Farace F. Detection of Gene Rearrangements in Circulating Tumor Cells: Examples of ALK-, ROS1-, RET-Rearrangements in Non-Small-Cell Lung Cancer and ERG-Rearrangements in Prostate Cancer. In: Magbanua MJM, Park JW, eds. *Isolation and Molecular Characterization of Circulating Tumor Cells*. Vol 994. Advances in Experimental Medicine and Biology. Springer International Publishing; 2017:169-179. doi:10.1007/978-3-319-55947-6_9

33. Novaes LAC, Sussuchi da Silva L, De Marchi P, et al. Simultaneous analysis of ALK, RET, and ROS1 gene fusions by NanoString in Brazilian lung adenocarcinoma patients. *Transl Lung Cancer Res*. 2021;10(1):292-303. doi:10.21037/tlcr-20-740

34. Lu H, Xu H, Xie F, et al. 1p/19q codeletion and RET rearrangements in small-cell lung cancer. *OncoTargets Ther*. Published online June 2016:3571. doi:10.2147/OTT.S108781

35. Michels S, Scheel AH, Scheffler M, et al. Clinicopathological Characteristics of RET Rearranged Lung Cancer in European Patients. *J Thorac Oncol*. 2016;11(1):122-127. doi:10.1016/j.jtho.2015.09.016

36. Lee MS, Kim RN, I H, et al. Identification of a novel partner gene, *KIAA1217* , fused to *RET* : Functional characterization and inhibitor sensitivity of two isoforms in lung adenocarcinoma. *Oncotarget*. 2016;7(24):36101-36114. doi:10.18632/oncotarget.9137

37. Sokolova IA, Bedroske P, Grushko TA, et al. Abstract 4256: Multiplex fast FISH assay for detecting ROS1, RET and MET aberrations in FFPE specimens using BioView image analysis. *Cancer Res*. 2020;80(16 Supplement):4256. doi:10.1158/1538-7445.AM2020-4256

38. Uguen M, Dewitte JD, Marcorelles P, et al. Asbestos-related lung cancers: A retrospective clinical and pathological study. *Mol Clin Oncol*. 2017;7(1):135-139. doi:10.3892/mco.2017.1277

39. Prager GW, Unseld M, Waneck F, et al. Results of the extended analysis for cancer treatment (EXACT) trial: a prospective translational study evaluating individualized treatment regimens in oncology. *Oncotarget*. 2019;10(9):942-952. doi:10.18632/oncotarget.26604

40. Tang Z, Zhang J, Lu X, et al. Coexistent genetic alterations involving ALK, RET, ROS1 or MET in 15 cases of lung adenocarcinoma. *Mod Pathol Off J U S Can Acad Pathol Inc*. 2018;31(2):307-312. doi:10.1038/modpathol.2017.109

41. Subbiah V, Berry J, Roxas M, et al. Systemic and CNS activity of the RET inhibitor vandetanib combined with the mTOR inhibitor everolimus in KIF5B-RET re-arranged non-small cell lung cancer with brain metastases. *Lung Cancer*. 2015;89(1):76-79. doi:10.1016/j.lungcan.2015.04.004

42. Zhang X, Li Y, Liu C, et al. Identification of a novel KIF13A-RET fusion in lung adenocarcinoma by next-generation sequencing. *Lung Cancer*. 2018;118:27-29. doi:10.1016/j.lungcan.2017.08.019

43. Mizukami T, Shiraishi K, Shimada Y, et al. Molecular Mechanisms Underlying Oncogenic RET Fusion in Lung Adenocarcinoma. *J Thorac Oncol*. 2014;9(5):622-630. doi:10.1097/JTO.0000000000000135

44. Lee SH, Lee JK, Ahn MJ, et al. Vandetanib in pretreated patients with advanced non-small cell lung cancer-harboring RET rearrangement: a phase II clinical trial. *Ann Oncol*. 2017;28(2):292-297. doi:10.1093/annonc/mdw559

45. Kohno T, Ichikawa H, Totoki Y, et al. KIF5B-RET fusions in lung adenocarcinoma. *Nat Med*. 2012;18(3):375-377. doi:10.1038/nm.2644

46. Offin M, Guo R, Wu SL, et al. Immunophenotype and Response to Immunotherapy of *RET* -Rearranged Lung Cancers. *JCO Precis Oncol*. 2019;(3):1-8. doi:10.1200/PO.18.00386

47. Platt A, Morten J, Ji Q, et al. A retrospective analysis of RET translocation, gene copy number gain and expression in NSCLC patients treated with vandetanib in four randomized Phase III studies. *BMC Cancer*. 2015;15(1):171. doi:10.1186/s12885-015-1146-8

48. Drilon A, Wang L, Hasanovic A, et al. Response to Cabozantinib in Patients with RET Fusion-Positive Lung Adenocarcinomas. Cancer Discov. 2013;3(6):630-635. doi:10.1158/2159-8290.CD-13-0035

49. Drilon A, Wang L, Arcila ME, et al. Broad, Hybrid Capture–Based Next-Generation Sequencing Identifies Actionable Genomic Alterations in Lung Adenocarcinomas Otherwise Negative for Such Alterations by Other Genomic Testing Approaches. Clin Cancer Res. 2015;21(16):3631-3639. doi:10.1158/1078-0432.CCR-14-2683

50. Yoh K, Seto T, Satouchi M, et al. Vandetanib in patients with previously treated RET-rearranged advanced non-small-cell lung cancer (LURET): an open-label, multicentre phase 2 trial. Lancet Respir Med. 2017;5(1):42-50. doi:10.1016/S2213-2600(16)30322-8

51. Lee J, Ku BM, Shim JH, et al. Characteristics and outcomes of RET-rearranged Korean non-small cell lung cancer patients in real-world practice. Jpn J Clin Oncol. 2020;50(5):594-601. doi:10.1093/jjco/hyaa019
